# Serum Sulphate Levels in Hemodialysis Patients

**DOI:** 10.1155/2019/1063514

**Published:** 2019-12-01

**Authors:** Ibrahim Yildirim, Ender Hur, Kemal Magden, Sevil İlikhan, Hüseyin Engin, Murat Can, Gürsel Yıldız, İsmail Özer

**Affiliations:** ^1^University of Health Sciences, Department of Internal Medicine, Division of Nephrology, Ankara, Turkey; ^2^Bulent Ecevit University Medical School, Department of Internal Medicine, Zonguldak, Turkey; ^3^Bulent Ecevit University Medical School, Department of Medical Biochemistry, Zonguldak, Turkey; ^4^Zonguldak Atatürk State Hospital, Department of Nephrology, Zonguldak, Turkey; ^5^Samsun Educational and Research Hospital, Department of Nephrology, Samsun, Turkey

## Abstract

**Objective:**

Sulphur, similar to phosphorus, is easily attached to organic compounds. The inadequate elimination of sulphate may cause high sulphate concentrations in hemodialysis (HD) patients because sulphate is low in free form in plasma. Although we are well aware of the accumulation of phosphorus in chronic dialysis patients, we do not have an adequate knowledge database about the sulphur compounds. This study was designed to determine the level of sulphate in hemodialysis patients.

**Materials and Methods:**

Ninety-four prevalent HD patients and 33 patients without renal failure were included in the study. The serum inorganic sulphate levels were measured by turbidimetric technique. Moreover, the serum level of urea, creatinine, albumin, calcium, phosphorus, and parathyroid hormone concentrations was simultaneously recorded.

**Results:**

Mean levels of plasma sulphate were significantly higher (0.56 ± 0.17 mM vs 0.31 ± 0.13 mM, *p* < 0.001) in HD patients. Serum sulphate level correlated with patient's age, serum albumin, serum BUN and creatinine, and serum phosphorus level in HD patients. Serum sulphate levels were not associated with serum parathyroid hormone levels.

**Conclusion:**

Serum sulphate levels were approximately twofold higher in HD patients than in the normal control group. Inorganic sulphate does not seem to accumulate in long-term dialysis patients, and mild increased serum levels of sulphate has no poor clinical outcome in these patients.

## 1. Introduction

Sulphur is an element which is abundant in the organism. Sulphur compounds are often present in the body as sulphur amino acids. A daily intake of inorganic sulphate is 0.2–1.5 g/day [1, 2]. The main sources of sulphur in the diet are inorganic sulphate and the sulphur-containing amino acids such as methionine, cystine, and cysteine. Some of the sulphate is a result of the metabolism of sulphur-containing proteins in the organism [2]. Metabolism of organic sulphur proteins supplies over half of the sulphate, and the remainder is supplied from organic sulphur-containing compounds or preformed sulphate in beverages and foods (beers, wines, fruits, some breads, soya flour, etc.) [3].

In the previous studies, normal inorganic sulphate levels have been reported as 0.32–0.39 mMol/L in healthy subjects [2–4]. Elevated serum sulphate concentrations can alter biochemical processes in the liver and lungs [5] and may disrupt the synthesis of glycosaminoglycan in the cartilage tissue [6]. Excessive intake of sulphate is associated with diarrhea [7] and ulcerative colitis [8]. In blood, high level of the sulphur compound is also associated with nephrolithiasis in healthy individuals [3]. It is also associated with uremic acidosis and bone disease in patients with renal failure [9]. It is considered by some authors as uremic toxins [10].

Due to its excretion from the kidney, sulphur compounds are accumulated in renal failure [3]. Plasma sulphate may be elevated up to 10 times the normal value in not on dialysis patients with a glomerular filtration rate (GFR) below 30 ml/min per 1.73 m^2^ [9]. Although the chronic effects of phosphorus accumulation are well known, there are limited studies on sulphate compound accumulation in dialysis patients [11–14] and most of these trials have a small number of patients. Although hemodialysis treatment removes sulphate [3, 12], there have been several studies demonstrating plasma sulphate concentrations do not fall to normal in serum, despite effective hemodialysis and peritoneal dialysis: [2, 4, 15]. Inorganic SO_4_ levels have been reported to be 5 times higher in dialysis patients than healthy subjects [16]. Some studies have also shown that increased plasma sulphate concentration gives rise to increased complexation with calcium. Therefore, it has been reported that “this may be partially responsible for the parathyroid stimulation that occurs in chronic renal disease” [3, 11]. For these reasons, SO_4_ clearance may be a problem in patients on maintenance hemodialysis (HD).

Sulphur is one of the anions that is not measured in the routine examinations, and this element has a high degree of protein binding, which limits dialysis clearance [3]. Its accumulation in dialyzed patients remains controversial. Moreover, the relationship between the dialysis duration and the accumulation of sulphate was not investigated previously. If sulphate compounds accumulated in dialyzed patients, they might lead to cartilage and bone diseases like renal osteodystrophy or to other disorders over a period of years. The clinical effects of high inorganic sulphate levels are not well known in HD patients. The objective of this study was to determine the level of inorganic sulphate accumulation in patients who receive HD with a synthetic dialyzer and to evaluate the association between sulphate accumulation and the duration of hemodialysis which was calculated in years from the start of hemodialysis therapy.

## 2. Materials and Methods

After an approval of the Ethics Committee of Bulent Ecevit University, a total of 127 participants were included in this study. All patients with end-stage renal disease (ESRD) were recruited from the patients undergoing maintenance HD from the dialysis centers in Zonguldak, Turkey. Study participants were selected according to nonprobability and purposive sampling techniques. The total number of HD patients in the Zonguldak provincial center was 584 at that period. Patients older than 18 years, who were willing to participate in the study and signed a written informed consent, and who were on maintenance HD therapy scheduled thrice weekly (a treatment time of 4 hours per session) for 3 months or longer were included in the study. Patients included in this trial had a urine output of less than 100 ml/day. A total of 33 individuals without renal failure were included in the control group. Exclusion criteria were the permanent or temporary catheters, presence of serious life-limiting comorbid conditions (e.g., malignancy, uncontrollable infection, malnutrition, and end-stage cardiac, pulmonary, or hepatic disease), and being pregnant or lactating. Four dialysis patients were excluded from this study because they had significant residual renal function, and so our study was performed in ninety HD patients and thirty-three controls.

All participants had the following data: age, gender, body mass index (BMI), comorbid diseases, taking medication, and e-GFR (in the control group). Patients' blood samples were obtained as part of the routine monthly monitoring. Fasting blood samples were drawn from the antecubital veins of the control group, and predialysis blood samples were drawn from the arteriovenous fistula of HD patients after the long interdialytic interval. Our patients were studied for 72 hr after their last hemodialysis. Biochemical findings such as urea, creatinine, albumin, calcium, phosphorus, parathormone (PTH), and complete blood count were measured at that time. The results of these biochemical markers were taken from HD patient's files. In patients without renal failure, these biochemical parameters were also measured. Obtained blood samples from the study population were stored at −80°C until the sulphate analysis. Subsequently, serum inorganic sulphate levels were measured with the quantitative turbidimetric sulphate specification kit (QuantiChrom ™ Sulphate Assay Kit (DSFT-200)). Serum PTH levels were measured with chemiluminescence method by using Abbott I 2000 (BIOKIT, S.A., Can Malé, s/n, 08186 Lliçàd'Amunt, Barcelona, Spain). PTH was measured with reference values of 15–84 pg/mL, and serum urea, creatinine, albumin, and phosphor levels were measured with spectrophotometric methods by ARCHITECT C 16000. Leukocyte count, platelet count, and hemoglobin concentrations were measured by using the Mindray BC6800 hematology analyzer.

All HD patients used synthetic dialyzers (80 patients with a surface area of 1.6 m^2^ and 14 patients with a surface area of 1.8 m^2^). All dialysates contained bicarbonate solution. The dialysate content was 1.25 mmol/l calcium, 0.5 mmol/l magnesium, 138–148 mmol/l sodium, 2 mmol/l potassium, 110 mmol/l chloride, 4.0 mmol/l acetate, and 33 mmol/l bicarbonate. The dialysate sulphate level was negligible (max 0.4 *μ*mol/l). An average blood flow rate was 300 ml/min, and dialysate flow rate was 500 ml/min.

The patients were divided into 4 groups according to HD vintage: group 1, those subjects with less than one year of dialysis time; group 2, those who incurred between 1 and 5 years on dialysis; group 3, those who received dialysis for 5 to 10 years; and group 4, those subjects with more than 10 years of dialysis times.

## 3. Statistics

Statistical analyses were performed by SPSS 18.0 software (SPSS Inc., Chicago, IL, USA). Distribution of data was determined by Kolmogorov–Smirnov test. Continuous variables were expressed as median (minimum-maximum) and categorical variables as frequency and percent. Continuous variables were compared with the Mann–Whitney *U* test and categorical variables were compared using Pearson's chi-squared test. Linear relation between two continuous biochemical variables was evaluated by Spearman's correlation analysis. The results were evaluated at 95% confidence level, and a *p* value of less than 0.05 was considered statistically significant for all tests.

## 4. Results

No patients had significant residual renal function in the HD group. All individuals in the control group had an e-GFR above 60 ml/min per 1.73 m^2^. Moreover, no one had malnutrition in our study population. Comorbid conditions and demographics of all study participants are summarized in Table 1. There was no significant difference between both HD and control groups in the incidence of diabetes mellitus and hypertension, obesity (body mass index (BMI)), and the age and gender differences (*p* values >0.05).

Arithmetic means of biochemical parameters in HD and control groups are presented in Table 2.

As expected, the average of urea, creatinine, and phosphorus values was higher in the HD group than the control group. There was no significant difference in plasma albumin concentrations between both the groups.

No significant difference in the serum inorganic sulphate (i-SO4) concentration was noted between female and male dialysis patients (male 0.57 vs female 0.54 mmol/L, *p*=0.79) and the control group (0.30 vs 0.32 mmol/L, *p*=0.35).

Of hemodialysis patients, 90% was taking oral phosphate binders. No difference was seen in plasma i-SO_4_ concentrations between patients taking oral phosphate binders and those of not taking [0.43 (0.33–0.88) mmol/L and 0.56 (0.16–0.92) mmol/L, respectively, *p*=0.44].

The calcium-sulphate interaction might be as significant as the calcium-phosphate interaction in patients with chronic renal failure (CRF) [2]. In a study, administration of sulphate resulted in elevated release of mineral from the bone in rats [17]. Another study suggested that the increased sulphate-calcium complexation might contribute to the parathyroid stimulation that occurs in chronic kidney disease [18]. In our trail, the median levels of serum PTH in the HD patients were 487 pg/ml (min: 110–max: 1684). Plasma PTH levels and i-SO_4_ were not correlated (Table 3).

Plasma sulphate level in the HD group correlated negatively with age and positively with plasma urea, creatinine, phosphorus, and albumin levels. A relation was not found between dialysis duration and plasma sulphate level either (Table 3).

**Figure 1:**
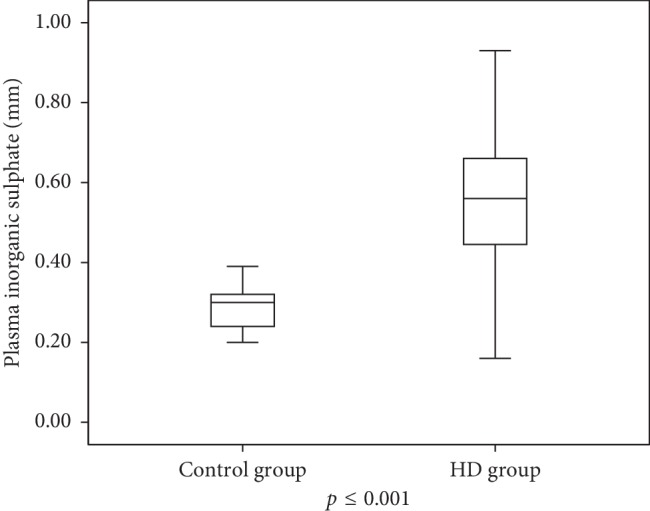
Mean plasma sulphate concentration in patients with renal failure and without renal failure (control group).

The median levels of serum i-SO_4_ in the HD patient group was approximately two times higher than that of the control group [0.53 (0.04–0.93) vs 0.3 (0.11–0.86) mMol/L, *p* < 0.001] (Figure 1).

**Figure 2:**
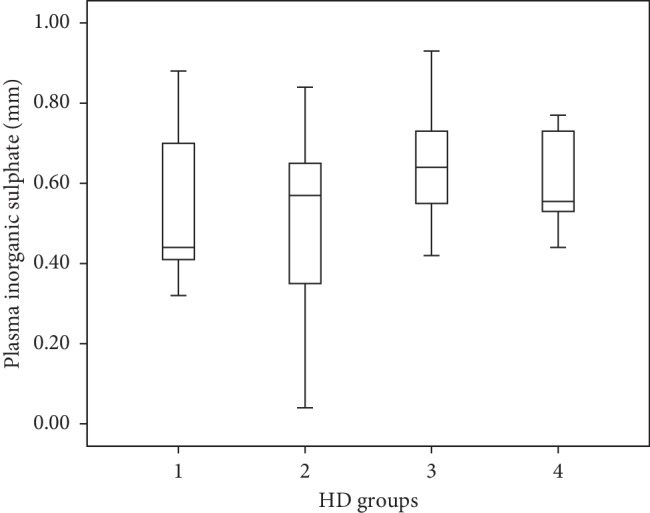
Serum inorganic sulphate scatter chart by year in patients on hemodialysis. Group 1, patients with less than one year of dialysis time; group 2, patients who incurred between 1 and 5 years on dialysis; group 3, patients who received dialysis for 5 to 10 years; and group 4, patient subjects with more than 10 years of dialysis times.

The median duration of dialysis was 4 years (min: 0.1–max: 25 years). There was no difference between the dialysis groups in terms of dialysis adequacy (*p* = 0.167). Plasma inorganic sulphate levels were not different in the HD groups based on dialysis duration (*p* = 0.216). The median values are presented in Table 4, and the correlation graph is shown in Figure 2.

## 5. Discussion

This article is one of the rare studies conducted to evaluate the issue of sulphate accumulation in patients with ESRD. The number of patients in our study was approximately three times higher than in the other previous studies. In addition, most of the previous studies were conducted by using a nonsynthetic dialyzer. In this study, synthetic dialyzers were used in all HD patients. Sulphate accumulation in nondialyzed patients with CRF has been known with certainty [3], but its accumulation in dialyzed patients remains controversial. In our study, the mean serum level of sulphate was significantly higher in HD patients, but it was not associated with serum parathyroid hormone levels.

In the previous studies, it was reported that increased sulphate levels might lead to bone diseases in patients with ESRD. Marangella et al., reported that increased sulphate levels could have adverse effects on bone turnover [2], Apella and Baran reported that this compound might substitute for phosphate in the apatite matrix in the bone [19], and Freeman and Richards reported that alkaline phosphatase levels were higher in the high-sulphate group [4]. In our study, there was no correlation between corrected serum calcium level and serum inorganic sulphate level in the HD patient group. Two studies have pointed out that increasing serum sulphate levels depresses the ionized calcium concentration [20, 21]. One of these studies was an animal study, and it was performed in rats without CRF [20]. In the other study of 9 patients who received sodium sulphate infusions, the authors observed a decrease in mean ionized calcium. These patients also have no CRF [21]. Therefore, the relationship between serum calcium and inorganic sulphate level is not clear. Moreover dialysis treatment could restore the distribution of the calcium salts to normal values.

We could not demonstrate any significant correlation between sulphate and serum phosphorus levels both in the HD patient group and in the control group either. A positive correlation with phosphorus and sulphate was emphasized in the previous two studies conducted in patients with stage 1 to 4 chronic kidney disease [11, 20]. Because both minerals are excreted by the kidney, in renal failure, sulphate and phosphorus retention can be misleading. There is no study evaluating the phosphorus-sulphate level correlation in a population without renal failure. We concluded that serum phosphorus and serum sulphate levels are independent of each other. Michalk et al. mentioned that the increased sulphate was accompanied by greater complexation with calcium, which might contribute to the parathyroid stimulation that occurs in chronic kidney disease [18]. There was no significant correlation between parathyroid hormone levels and sulphate levels in the HD patient group in our study.

Serum inorganic sulphate level was correlated negatively with age in both the HD patients and control group in the present study. As the patient gets older, both dietary intake and gastrointestinal absorption of foods containing sulphur were decreasing [3]. In this literature, a negative correlation was also reported between age and serum i-SO_4_ levels in individuals without renal failure. Aging-related decline in GFR requires a strong positive correlation between sulphate levels and renal function. Cole and Evrovski emphasized a correlation with diet [3], but in this literature, all participants with kidney disease were patients with stage 1–4 renal failure and these patients were not receiving renal replacement therapy. Furthermore, the drugs used by renal patients that reduce phosphorus absorption may also reduce the absorption of sulphur compounds from the intestines. We have no data at this issue, but in our study, there was no significant difference in plasma i-SO_4_ concentrations between patients taking oral phosphate binders and those of not taking these drugs.

In the study by Suliman et al., serum sulphate levels were higher in male patients when compared to female patients on dialysis (1.59 vs 1.11 mmol/L) [16], but the levels in the control group were similar in both sexes. In one study, there was no difference between serum sulphide levels and patient's age and gender in the healthy control group (*n* = 20) and predialysis patient group (*n* = 33) [22]. We found no gender difference in the levels of serum i-SO_4_ in both the HD and control group.

There are few studies demonstrating an association between serum sulphate and albumin levels in HD patients. Brunetti et al., found a significant positive correlation in their study of 18 HD patients [13]. In our study, plasma sulphate levels were weakly positively correlated with the serum albumin and urea concentration in the HD patient group. Serum sulphate levels were also correlated with serum blood urea nitrogen in the control group. This correlation was mostly related to BMI of dialysis patients. The correlation between serum sulphate levels and BMI may be because larger people eat more food and therefore eat more sulphate.

Kirchbaum mentioned that the sulphate reduction ratio exceeded the urea reduction ratio, but plasma sulphate levels could often still be elevated [12]. In the present study, the plasma inorganic sulphur levels were 1.8 times higher in HD patients when compared with thosewithout renal failure (0.56 ± 0.17 mM vs. 0.31 ± 0.13 mM, *p* < 0.001). Our results were consistent with those of a study by Freeman and Richards [4], but the plasma sulphate levels were lower in our HD patients. In the study by Freeman et al., the mean inorganic sulphate level in HD patients was 2.07 ± 0.13 mmol/l. Serum i-SO_4_ levels in 18 HD patients were 5 times higher than healthy individuals in another study (1.44 mmol/L to 0.31 mmol/L) [4]. In our study, a reason for the lower serum inorganic sulphate levels in HD patients has been thought to be due to the more effective sulphate clearance with a synthetic dialyzer.

In our study, we evaluated whether or not the serum inorganic sulphate concentration increased with the duration of dialysis. We suspected if clearance of sulphur was reduced by dialysis, it could have accumulated in patients who had been receiving dialysis over a long period of time. But we could not demonstrate the accumulation of sulphate in different four groups based on the duration of HD.

In conclusion, serum i-SO_4_ can be readily cleared by synthetic dialyzers. We believe that serum inorganic sulphate, unlike phosphorus, does not accumulate in long-term dialysis patients.

## Figures and Tables

**Table 1:** Clinical characteristics of study participants.

	HD group (*n* = 90)	Control group (*n* = 33)	*p* value
Mean age (min-max)	57.8 (22–88)	63.3 (25–82)	0.079
Gender (F/M) (*n*)	30/60	13/20	0.104
Diabetes mellitus (*n*)	37 (41%)	13 (39%)	0.077
Hypertension (*n*)	58 (64%)	19 (57%)	0.061
Ischemic cardiac disease (*n*)	40 (44%)	6 (18%)	0.052
Median BMI (min-max)	26.5 (22–36)	26 (22–33)	0.288

F: female, M: male, BMI: body mass index, min: minimum, and max: maximum.

**Table 2:** Laboratory findings of study participants.

Plasma concentrations	HD group	Control group	*p* value
Urea (mg/dl)	134 (±30.84)	20 (±11.64)	<0.001
Creatinine (mg/dl)	8.3 (±3.67)	0.8 (±0.37)	<0.001
Albumin (g/dl)	3.5 (±0.34)	3.5 (±0.58)	0.756
CCa (mg/dl)	8.9 (±0.68)	9.1 (±0.78)	0.035
Phosphorus (mg/dl)	5.4 (±1.41)	3.7 (±0.85)	0.002
Parathormone (pg/ml)	554 (±355)	48 (±30.47)	<0.001
i-SO_4_ (mmol/l)	0.56 (±0.17)	0.31 (±0.13)	<0.001

CCa: corrected calcium. Results are given as mean ± standard deviation.

**Table 3:** Clinical and biochemical correlations of plasma i-SO_4_ level in the HD group.

	*r*	*p* value
Age (years)	−0.303	0.004
Urea (mg/dl)	0.459	<0.001
Alb (g/dl)	0.23	<0.001
CCa (mg/dl)	−0.128	0.225
Phosphorus (mg/dl)	0.206	0.050
HD time (months)	0.224	0.060
PTH (pg/ml)	0.154	0.187

Alb: serum albumin concentration; CCa: corrected calcium; HD time: the duration of hemodialysis which was calculated in years from the start of hemodialysis therapy; PTH: parathormone; Hb: hemoglobin.

**Table 4:** Hemodialysis patient groups based on the duration and inorganic sulphate levels.

HD group	pSO4 level medians	Min–max
Group 1 (*n* = 14)	0.5393 (0.44)	0.32–0.88
Group 2 (*n* = 32)	0.525 (0.57)	0.04–0.84
Group 3 (*n* = 15)	0.6413 (0.64)	0.42–0.93
Group 4 (*n* = 10)	0.596 (0.555)	0.04–0.93

## Data Availability

The data that support the findings of this article are available from the corresponding author (IY), upon reasonable request, via breibrahim@yahoo.com.

## Additional Points

*Study Limitations*. When creating study population, we took into account some circumstances that would affect our results. Namely, it was known that inorganic sulphate levels were low in patients with protein malnutrition [16], so HD patients with good nutritional status were included in our study. In addition, our study was performed only in hemodialysis patients because the type of dialysis was affecting sulphate clearance. Previous studies have shown that serum i-SO_4_ levels are lower in peritoneal dialysis patients compared with HD patients [16].
